# Diagnostic Value of Conventional Polymerase Chain Reaction for Detecting BRAF V595E Mutation in Liquid and Tissue Specimens of Canine Urothelial and Prostate Carcinomas

**DOI:** 10.3390/ani14172535

**Published:** 2024-08-31

**Authors:** Chien-Chun Kuo, Su-Ya Yang, Ru-Min Liu, Yung-Hsuan Lin, Chih-Chun Liu, Wei-Hsiang Huang, Jih-Jong Lee, Albert Taiching Liao

**Affiliations:** 1 Department of Veterinary Medicine, School of Veterinary Medicine, National Taiwan University, No. 1, Sec. 4, Roosevelt Rd., Taipei City 106, Taiwan; d08629001@ntu.edu.tw (C.-C.K.);; 2 Graduate Institute of Molecular and Comparative Pathobiology, National Taiwan University, No. 1, Sec. 4, Roosevelt Rd., Taipei City 106, Taiwan; whhuang@ntu.edu.tw; 3 Graduate Institute of Veterinary Clinical Science, School of Veterinary Medicine, National Taiwan University, No. 1, Sec. 4, Roosevelt Rd., Taipei City 106, Taiwan; jacklee@ntu.edu.tw; 4 National Taiwan University Veterinary Hospital, College of Bioresources and Agriculture, National Taiwan University, No. 153, Sec. 3, Keelung Rd., Taipei City 106, Taiwan

**Keywords:** urothelial carcinoma, prostatic carcinoma, BRAF, conventional PCR

## Abstract

**Simple Summary:**

Urothelial carcinoma (UC) and prostatic carcinoma (PC) are common lower urinary tract cancers in dogs. The detection of the BRAF V595E gene mutation in urine samples could provide a non-invasive method for diagnosing these cancers. However, the established method requires a specialized machine, which is not available at every diagnostic center. In this research, we established a simple, low-cost, conventional PCR method to the detect gene mutation indicative of UC and PC. The results showed that this conventional PCR method could provide a reliable, non-invasive diagnostic tool, aiding in the clinical diagnosis of UC and PC in dogs, especially in settings without advanced PCR equipment.

**Abstract:**

Canine urothelial carcinoma (UC) and prostatic carcinoma (PC) often present diagnostic challenges due to their anatomical locations. The BRAF V595E mutation, analogous to the human BRAF V600E mutation, has been identified in UC and PC. Digital PCR of urine is a non-invasive diagnostic method of mutation detection, but the availability of the necessary equipment is limited. This study aimed to develop a conventional PCR to detect the BRAF V595E mutation in urine and prostatic wash specimens from dogs with UC or PC. Specific primers for detecting wild-type and mutant BRAF V595E genes were validated in 34 formalin-fixed paraffin-embedded (FFPE) tissues, 116 urine samples, and 9 prostatic wash specimens. The results showed that the BRAF V595E mutation detection rate for UC and PC in the tissues was 51.6%. The detection rate in liquid specimens from dogs with lower urinary tract or prostate masses was 53.2%. Of the 41 cases with follow-up, 16 were further diagnosed with UC or PC, with 75% of liquid specimens from these dogs showing the BRAF V595E mutation. This conventional PCR method provides a reliable and non-invasive screening tool for UC and PC in dogs, especially in settings without advanced equipment.

## 1. Introduction

Urothelial carcinoma (UC), or transitional cell carcinoma (TCC), is a common urinary tract tumor in dogs. UC frequently occurs in the trigone area but can also invade or independently occur in the urethra and prostate [1,2,3]. Female dogs are at a higher risk [2], and high-risk breeds include Scottish Terriers, Eskimo Dogs, Shetland Sheepdogs, and West Highland White Terriers [2,4]. UC is considered to be a spontaneous animal cancer model for human bladder cancer, as it exhibits similar pathological features [2,3,5,6]. Most UCs are of pathologically intermediate to high grade, exhibiting aggressive behavior with potential for metastasis to the regional lymph nodes, skin, and lungs [1,2,4]. The clinical signs of UC are similar to those of urinary tract infections (UTIs) and may present with polyuria and hematuria [4]. The diagnostic examination often includes ultrasonography, cytology of the urinary sediment, and histopathology [4]. Cytological examination is a non-invasive diagnostic method, but UC commonly coexists with urinary tract infections in which atypical epithelial cells may be observed in the urinary sediment [7].

The diagnosis of UC relies on a histopathological exam of tissue obtained via cystoscopy or surgery [1,8], which could be challenging when the mass is located in the trigone area. Similarly, prostate carcinoma (PC) presents diagnostic challenges due to its anatomic location, where the tumors cannot be easily reached. PC may originate from either urothelial or glandular epithelium [9,10]. In addition, urothelial carcinoma of the prostatic urethra frequently invades the prostate, making it difficult to distinguish from prostate epithelial cancer [10]. Although histopathology is the gold standard for diagnoses, the anatomical location often complicates sampling, leading to reliance on the cytology of prostatic wash fluids for diagnosis [11]. When the tumor’s location makes surgical removal difficult, chemotherapy or non-steroidal anti-inflammatory drugs (NSAIDs) may be administrated before a histopathological diagnosis [1,12].

Over the last decade, studies have reported the BRAF V595E mutation in both UC and PC [13,14,15,16]. BRAF, as a serine/threonine kinase, is overactivated by a point mutation, inducing the RAS-RAF-MEK-ERK-MAP kinase pathway and leading to cancer’s proliferation [17,18,19]. This mutation occurs at position 1394 of the BRAF gene, involving a single-nucleotide substitution [14,15]. The nucleotide substitution from adenine (A) to thymine (T) results in the replacement of valine (V) with glutamic acid (E) at codon 595 of the canine BRAF kinase (V595E, according to the canine BRAF reference sequence: Ensembl Transcript ID: ENSCAFT00000006306). This mutation is homologous to the human BRAF V600E mutation [13,14,17]. About 15–30% of human tumors harbor BRAF mutations, predominantly in 30–60% of malignant melanomas in humans [17,19]. However, BRAF mutations do not predominate in canine melanomas [14]. Other canine tumors exhibiting the BRAF V595E mutation include pulmonary carcinoma, oral squamous cell carcinoma, melanoma, glioma, and peripheral nerve sheath tumor, but their mutation rates are less than 25% [14].

Various methods have been established to detect the BRAF V595E mutation, including PCR with Sanger sequencing [13,14,15], quantitative PCR [20], droplet digital PCR (ddPCR) [15,21], and digital PCR [22]. These techniques have detected the mutation in different types of specimens, including blood [14,20], fresh tissues [13,14], formalin-fixed paraffin-embedded (FFPE) tissues [13,14], and urine specimens [13,15,22]. Studies have reported that the mutation rate is approximately 67% to 80% in UC [13,14,15] and about 79% to 85% in PC [14,15]. The mutation rate in urine from dogs with UC ranges from 50% to 83% [13,15,22], and the detection rate in urine from dogs with PC is 100% [15]. Therefore, the use of urine or prostatic wash fluids to detect the presence of the BRAF V595E mutation has been developed as a non-invasive method to diagnose UC and PC [13,15]. The sensitivity of detecting BRAF mutations is related to the detection method. Conventional PCR combined with Sanger sequencing requires 15–20% mutant alleles for the reliable detection of single-nucleotide substitutions [23].

Currently, digital PCR is regarded as a standard tool for detecting mutations in liquid specimens because its sensitivity is higher than that of conventional PCR [15,21]. Despite its higher sensitivity, digital PCR requires specific PCR equipment that may not be available in diagnostic facilities. Therefore, this study aimed to establish a conventional PCR method as a screening tool to detect the BRAF V595E mutation in urine or prostatic wash specimens from dogs with UC or PC.

## 2. Materials and Methods

### 2.1. Study Design

In this study, two sets of primers were used to detect the BRAF wild-type gene and the mutated BRAF V595E gene using conventional PCR. Initially, this PCR method was validated to detect mutant genes in FFPE tissues and urine specimens of UC. Subsequently, this conventional PCR method was applied to evaluate the BRAF V595E mutation rate of UC and PC in FFPE tissues.

Urine or prostate wash specimens were collected from patients to detect the mutant gene. These patients, exhibiting urinary tract or prostate masses, were screened for the possibility of cancer on the basis of the BRAF mutation. After detection of the BRAF V595E mutation in the liquid specimens, a histopathological or cytological examination was recommended by their clinical veterinarians.

To evaluate the clinical diagnosis and implications of this conventional PCR screening test, a 1-year follow-up of patient outcomes was conducted. This study included patients who received a pathological diagnosis and those who did not. For patients with a pathological diagnosis, the mutation detection rate in fluid specimens was evaluated. For patients who did not undergo further pathological exams, the disease outcomes were assessed based on the evaluation of mass lesion by clinical veterinarians.

### 2.2. Tumor Tissues

Formalin-fixed paraffin-embedded (FFPE) UC and PC tissues were retrieved from the histopathological tissue archive of the Graduate Institute of Molecular and Comparative Pathobiology (GIMCP), National Taiwan University (NTU). In total, 34 tissue blocks, which were diagnosed as UC (*n* = 32) or PC (*n* = 2) between April 2019 and December 2022, were included in this study. From each block, 8-micrometer tissue sections were used for DNA extraction. Genomic DNA was extracted from the FFPE tissue using the QIAamp^®^ DNA FFPE Advanced Kit (Qiagen, Hilden, Germany), according to the manufacturer’s instructions. The DNA was eluted with nuclease-free water, and the quality was measured using a Nanophotometer^®^ (Implen, Munich, Germany).

### 2.3. Conventional PCR

Two sets of primers, modified from a previous study [20], were used to amplify fragments of the BRAF gene located on canine chromosome 16. These primers could amplify fragments of both the wild-type and mutant genes, each 138 bp in size (CanFam 3.1, canine chromosome 16: 8003308-8003445). The amplified sequence was designed using Primer-BLAST software (Primer3, version 2.5.0). The primer pairs listed in Table 1 were used for PCR to detect the wild type (Wt) and V595E mutant type (Mu) of the BRAF gene. PCR using the GAPDH primer pair was used as the internal control. Each PCR reaction included 200 ng of genomic DNA, 500 nM of the forward and reverse primers, and Taq DNA Polymerase 2x Master Mix RED (Ampliqon, Odense M, Denmark). An initial denaturation for 2 min at 95 °C was followed by 35 thermal cycles of denaturation for 30 s at 95 °C, annealing for 30 s at 62 °C, and extension for 30 s at 72 °C. A final elongation step at 72 °C for 5 min was conducted at the end of the thermal cycle.

Genomic DNA from the UCDK9M1 cell, a canine melanoma cell line, was used as the wild-type control of BRAF. PCR products were identified using 3% agarose gel electrophoresis. To verify the sequence, PCR products were subjected to targeted Sanger sequencing analysis with the forward and/or reverse primers, performed by the DNA Sequencing Core of the Center for Biotechnology, National Taiwan University. Sequence analysis was performed at the appropriate sequencing facility and analyzed using 4Peaks software (version 1.8, Nucleobytes, Amsterdam, The Netherlands).

### 2.4. Liquid Specimens from Dogs with Lower Urinary Tract or Prostate Masses

Urine or prostatic wash specimens from dogs suspected of having urinary tract masses or an enlarged prostate, presenting at National Taiwan University Veterinary Hospital (NTUVH) and regional veterinary hospitals between July 2021 and November 2023, were submitted to the laboratory for detection of the BRAF mutation. The clinical indication for submission was based on the findings of clinical examinations, such as irregular bladder wall lesions, a bladder wall mass, or prostatic enlargement observed via ultrasonography, to screen for the possibility of UC or PC.

The specimens, which included urine and prostatic wash fluids, were collected at a recommended volume of 20 mL, transported, and stored at 4–8 °C. They were processed within 3 days for total DNA extraction. Total DNA was extracted by centrifuging the urine or prostatic wash fluid at 2000 rpm for 10 min at 4 °C, discarding the supernatant, resuspending the pellets in 200 μL PBS, and employing the DNeasy^®^ Blood and Tissue Kit (Qiagen, Hilden, Germany), according to the manufacturer’s instructions. The DNA was eluted with nuclease-free water, and the quality was measured using a Nanophotometer^®^ (Implen, Munich, Germany).

### 2.5. Patients to Be Followed Up Regarding the Clinical Outcome

This study retrospectively included dogs suspected of having urinary tract or prostate tumors, and the BRAF mutation test was performed on their liquid specimens at NTUVH. Ultrasonographic examinations of these dogs showed bladder masses, irregular bladder walls, or prostate enlargement. The method of detecting the BRAF mutation was described in Section 2.4. Urine sediments or prostatic wash fluid specimens were collected for cytological examination and for detection of the BRAF mutation to diagnose UC or PC.

On the basis of the cytological findings and the results of the BRAF mutation detection, an additional histopathological examination was recommended for pathological diagnosis. The diagnosis of UC or PC was based on the cytological and histopathological diagnosis. Further treatment could include chemotherapy, non-steroidal anti-inflammatory drugs, surgical excision, castration, or routine monitoring.

For patients who were not diagnosed with cancer by a pathological exam after testing for the BRAF mutation, the outcome of the disease was observed after at least 6 months. To monitor the tumor’s progression, ultrasonographic examinations were performed for at least 6 months. Patients were classified as showing progressive of the disease or no progression, according to the size of the masses. Progressive disease was defined as a tumor with a diameter that increased by more than 30% of the original lesion size. Patients without progressive disease included those who showed a stable tumor size, a reduction in size, or complete remission without receiving treatment or undergoing castration, with no neoplastic cells confirmed via histopathology or cytology. Patients diagnosed with a urinary tract infection or benign prostatic hyperplasia could be included in this category. Other data collected included age, sex, neuter status, and breed.

The experimental protocol received ethical approval from the Institutional Animal Care and Use Committee (IACUC) of National Taiwan University under the IACUC number NTU-113-EL-00049. Canine patients whose urine or prostatic wash specimens were submitted for examination for the BRAF mutation at the National Taiwan University Veterinary Hospital (NTUVH) were included in the study.

## 3. Results

### 3.1. Detection of the BRAF V595E Mutation in the Tissues of Urothelial Carcinoma and Prostate Carcinoma

To confirm that the conventional PCR method could detect and differentiate the wild-type and mutated BRAF genes in tumor tissues, tumor tissues from six UC and two PC samples were collected from the FFPE tissue archive at the GIMCP of NTU. DNA was extracted from these tissues, and PCR was performed using the primer pairs listed in Table 1 to detect the wild-type and mutated BRAF genes in the tumor tissue.

**Figure 1:**
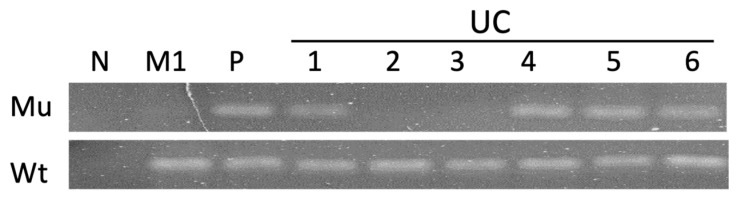
The representative PCR results of the formalin-fixed paraffin-embedded tissues from canine urothelial carcinoma (UC) samples. PCR tests using wild-type (Wt) and mutant (Mu) primer pairs were applied to detect the wild type and V595E mutant of the BRAF gene in the DNA extracted from six UCs (UC1 to UC6). DNA extracted from a melanoma cell (M1), which lacked the BRAF mutant gene, and double-distilled sterile water were used as the wild-type BRAF gene and negative control, respectively. DNA extracted from the urinary sediment of UC with the BRAF mutation was used as a positive control. The original electrophoresis image refers to Supplementary Figure S1.

The results demonstrated that the two primer pairs could detect and differentiate between the wild-type and mutant BRAF genes. Representative PCR results are shown in Figure 1. Using the mutant-specific primer pair, we detected the BRAF V595E mutation in one prostatic carcinoma sample (PC2) and four urothelial carcinoma tissues (UC1, UC4, UC5, and UC6). The BRAF V595E mutation was not detected in one prostatic carcinoma sample (PC1) and two urothelial carcinoma samples (UC2 and UC3). All PCR results using the wild-type primer pair successfully detected the BRAF gene, indicating that this primer could serve as an internal control in the subsequent experiments to validate the efficacy of the PCR method.

To explore the proportion of the BRAF V595E mutation in the UC and PC samples, we further collected 26 UC tissue blocks from the FFPE tissue archive and extracted DNA from these samples. Using the Wt and Mu primer pairs, we performed PCR to detect the BRAF V595E mutation. In total, 32 UC and 2 PC tissue blocks were analyzed. The results showed that 15 UC tissue samples and 1 PC tissue sample tested positive for the BRAF V595E mutation, while 14 UC tissue samples and 1 PC tissue sample did not harbor the BRAF V595E mutation. Three UC tissue samples did not produce any PCR products with either the Wt or Mu primer pairs; therefore, the BRAF mutation was considered undetectable in these samples. Excluding these three undetectable samples, the detection rates of the BRAF V595E mutation in UC and PC were 51.7% (15/29) and 50% (1/2), respectively. The overall rate of detection of the BRAF V595E mutation in urothelial tumors from the FFPE tissue was 51.6% (16/31), as summarized in Table 2.

### 3.2. Using Conventional PCR to Detect the BRAF V595E Mutation in the Urine and Prostatic Wash of Dogs with Lower Urinary Tract or Prostate Masses

**Figure 2:**
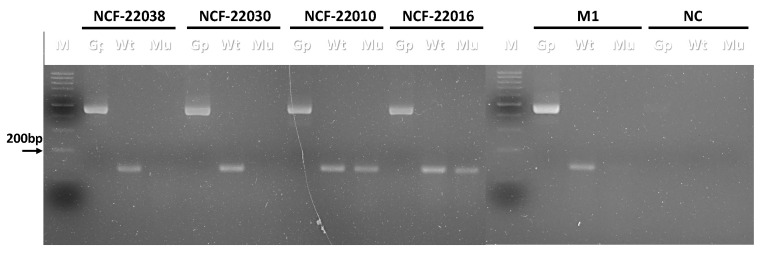
The representative PCR results of liquid specimens. Wild-type (Wt) and V595E mutant (Mu) PCRs were applied to detect the BRAF gene in the DNA extracted from the sediment of four urine samples (NCF-22038, NCF-22030, NCF-22010, and NCF-22016). GAPDH (Gp) was also applied as the internal control. DNA extracted from a melanoma cell (M1) and sterile double-distilled water (N) were used as the wild-type BRAF gene and the negative control, respectively. The size of the PCR products for GAPDH (Gp) and BRAF (Wt and Mu) were, respectively, 452 bp and 138 bp.

The conventional PCR method was verified to detect the BRAF V595E mutation in liquid specimens. Urine samples from dogs diagnosed with urothelial carcinoma were tested, and conventional PCR was used to detect both the wild-type (Wt) and mutant (Mu) BRAF V595E mutation in the urine samples, with GAPDH serving as an internal control. Representative results are shown in Figure 2, demonstrating that the internal control and BRAF wild-type genes were detected in all samples, while not all samples presented the BRAF V595E mutation. These results demonstrated that this conventional PCR method can detect the BRAF V595E mutant gene in liquid specimens.

In total, 125 urine or prostatic wash specimens were collected, including 116 urine and 9 prostatic wash specimens. These specimens were obtained from dogs suspected of having lower urinary tract or prostatic masses on the basis of an ultrasonographic examination at the National Taiwan University Veterinary Hospital and regional veterinary hospitals. The average volume of the liquid specimens was 32 mL, with amounts ranging from 7 to 100 mL.

The results indicated that the BRAF V595E mutation was found in 63 specimens, while 54 specimens did not harbor the BRAF V595E mutation. A total of 8 specimens (7 urine and 1 prostatic wash) possessed insufficient amounts of DNA for detection by PCR. Among the 117 specimens with sufficient DNA, the BRAF V595E mutation was detected in 54.1% (59/109) of urine specimens and 50% (4/8) of prostatic wash specimens. The overall positive rate of the BRAF V595E mutation was 53.2% (63/117). The results are summarized in Table 2.

### 3.3. Long-Term Follow-Up of Patients with or without the BRAF V595E Mutation

The medical records of 47 patients from the NTUVH were assessed. All of their liquid specimens underwent mutation tests. A total of 41 dogs were included in the subsequent analysis due to the loss of 6 dogs during follow-up. A total of 16 were diagnosed with UC or PC by means of a cytological or histopathological exam. However, 25 dogs were not diagnosed with cancer by means of the cytological exam and did not undergo a histopathological exam. The correlation between a BRAF mutation in the results and the presence of a progressive tumor in these patients was analyzed. The follow-up outcomes and the presence of the mutation in these dogs are presented in Table 3.

Of the 16 dogs diagnosed with UC or PC via a cytological or histopathological exam, all underwent a cytological examination. A total of 14 dogs were cytologically diagnosed with carcinoma, but 2 dogs were not. However, they were diagnosed with UC in a further pathological examination. Four dogs were pathologically diagnosed with UC. The BRAF V595E mutation was detected in liquid specimens from 12 dogs, while 4 dogs did not harbor the BRAF mutation. The BRAF V595E mutation rate was 75%, and the negative rate was 25%. In addition, in the four dogs that underwent both the cytological and histopathological exams, the BRAF V595E mutation was detected in both the liquid and tumor tissue samples in two cases, while the other two dogs did not harbor the BRAF V595E mutation in either the liquid or tissue samples. The diagnostic methods and results for the mutation in these patients are detailed in Table 4.

Of the 41 dogs, 25 were not diagnosed with cancer through cytological exams. None of them underwent histopathological exams. This group included 12 dogs with the BRAF V595E mutation and 13 without the BRAF V595E mutation in their liquid specimens. Among the 12 mutation-positive dogs, their tumor progressively enlarged during the follow-up period. Among the 13 mutation-negative patients, tumor progression was observed in 2 cases. Masses or lesions showing remission or that were stable in size were observed in 11 patients following neutering or antibiotic therapy, without antineoplastic treatment.

## 4. Discussion

This study used the conventional PCR method to detect the BRAF V595E mutation in tissue, urine, or prostatic wash specimens to assist in diagnosing UC or PC. The BRAF V595E mutation, a canine point mutation homologous to the human BRAF V600E mutation found in approximately 8% of human tumors, has been found in around 85% of PCs and 80% of UCs in canines [13,14]. Recently, digital PCR was applied to enhance the sensitivity of mutation detection in the urine specimens from cases of canine UC [13,15,22]. Since then, the detection of the BRAF V595E mutation in the urine of dogs with suspected UC and PC has become an important diagnostic tool. We established a conventional PCR without sequencing to replace digital PCR when digital PCR is not available. The results showed that the detection rate of this conventional PCR for detecting the mutation in liquid specimens of UC or PC was about 75%, indicating that this method can be regarded as a non-invasive screening test aiding in the diagnosis of UC or PC.

Conventional PCR with sequencing to detect the BRAF mutation has been documented in previous studies, which has since transitioned to digital PCR to increase the sensitivity for detecting mutations [13,15,21,22,24]. Since not all diagnostic laboratories have digital PCR machines, we used conventional PCR without sequencing to detect the BRAF V595E mutation. We used two primers for the conventional PCR to separately detect non-mutant and mutant BRAF genes. Our method could identify the two genotypes without gene sequencing, making it more convenient and fast than other convenient PCR. In addition, digital PCR is more sensitive, but may have a higher false positive rate, with one study reporting a specificity of 55% and a positive predictive value of 44% [21]. In dogs where the mutation was detected but no tumors were present, most (33 of 80 dogs) did not develop tumors during the study period, with a median observation period of 18 months. Although conventional PCR is less sensitive than digital PCR, it may deliver a lower false positive rate.

Different sample types, such as FFPE tissue or body fluid, exhibit varying rates of mutation detection. In our study, the detection rate of the mutation in FFPE UC tissue was 51.7%, which was lower than that obtained in previous studies using conventional PCR, with a rate of 67% to 80.3% [13,14,15], and ddPCR, with a rate of 75% [15]. The lower detection rate may be caused by several factors, including the detection limit of conventional PCR, the low mutation rates in our tissue samples, and insufficient DNA in the FFPE tissue. In the case of PC tissues, our mutation detection rate was 50%, which was lower than the 80% rate reported in previous studies using conventional PCR [14,15] and the 85% rate reported for ddPCR [15]. It is important to note that the small number of PC patients in our study may have contributed to statistical errors.

When considering the BRAF mutation test as a screening test, we collected fluid specimens from various patients, which resulted in a lower detection rate. In our study, the mutation detection rate in all the urine samples was 54.1%. This rate was similar to that found in previous studies, which reported detection rates of 50% to 61% using conventional PCR with gene sequencing [13,15], but it was lower than the 83% detection rate obtained using ddPCR [15] and the 90.9% detection rate using dPCR [22]. The detection rate in the prostatic wash specimens was 50%, which was lower than the 100% reported using PCR with sequencing and ddPCR [15]. The lower detection rate in dogs with lower urinary tract or prostate masses can be attributed to a variety of underlying diseases in the patients, who may exhibit UC, PC, cystitis, non-urothelial tumors, prostate hyperplasia, or no disease, significantly reducing the mutation detection rate.

Most dogs in our study population did not undergo histopathological exams after testing for the mutation due to the owner’s concern. Because these data are valuable, we evaluated the patients’ clinical outcomes, including those diagnosed with UC or PC and patients without a pathological diagnosis, employing at least a 6-month follow-up period. The mutation detection rate was 75% in the patients diagnosed with UC or PC, similar to the results of a previous study showing that the mutation rate in the urine of dogs with UC ranged from 50% to 83% [13,15,22], and was 100% in dogs with PC [15]. The detection rate of the mutation was 75%, which was lower than the 83% detection rate obtained using ddPCR [15] and the 90.9% detection rate via digital PCR [22]. Most patients with the mutation undergo progression of the disease, indicating that the positive result of this conventional PCR method could identify patients with UC or PC. This indicates that detecting the mutation in liquid specimens using this conventional PCR method can be regarded as a screening test to identify dogs with UC or PC.

In this study, about 25% of the liquid specimens from the UC or PC patients were negative for the BRAF mutation, which was similar to the rates encountered in previous studies [15,22]. Possible reasons include that the tumor did not have the BRAF mutation, or that the mutated genes were diluted, which reduced the ratio below the detection limit of conventional PCR. Histopathological exams or other developing non-invasive biomarkers, such as miRNA [25,26,27] and proteomics [28], could be considered to diagnose UC or PC in these dogs.

The results were consistent for patients who underwent both liquid and tissue tests for the mutation, indicating that our sampling and detection methods were appropriate. The consistency between the results from testing urinary sediment and tissue can vary with the detection method. Studies have reported an 89% consistency between DNA from urinary sediment and tumor tissue using restriction fragment length polymorphism analysis, a statistic that can be increased to 100% using next-generation sequencing [13]. Therefore, the detection method may significantly influence the results of detecting the BRAF V595E mutation.

The false-positive result was absent in this study, indicating that all patients with the detected mutation exhibited tumor diseases. However, the BRAF V595E mutation has been identified in non-tumorous preneoplastic lesions, such as dysplasia [21], and in canine flat urothelial lesions with atypia [29]. Additionally, the lower sensitivity of conventional PCR may contribute to the reduced false-positive rate. When mutation detection has been used as a routine diagnostic tool in high-risk breeds, there have been instances in which dogs initially showed false positives but later developed cancer. This suggests that BRAFV595E mutation detection might identify potential cancer risks at an early stage [21]. Long-term follow-up is still recommended when dogs display the BRAF V595E mutation, even without disease progression.

In this study, as in previous studies, we could not determine whether PC originated from the urothelial epithelium or the prostatic epithelium. However, recent research has utilized immunohistochemistry (IHC) to detect the mutant BRAF V595E protein in UC and PC tissues [30]. It was shown that 59% of UC cases and 65% of PC cases possessed mutant BRAF V595E protein. When the results were compared with those for digital droplet PCR, IHC staining demonstrated a sensitivity of 99% and a specificity of 100%, indicating the feasibility of using IHC to detect BRAF mutation. This study also confirmed that PC, whether derived from the urothelial epithelium or the prostatic glandular epithelium, could express the BRAF V595E mutation [30]. Therefore, the mutation rate of PC might not be affected by the cellular origin, e.g., from the prostate gland’s epithelium or the urothelial epithelium.

Few studies have found a correlation between the BRAF V595E mutation and the patients’ characteristics. As we know, terriers with UC may have a higher mutation rate than non-terrier breeds [31]. In addition, the BRAF V595E mutation does not seem to be associated with the prognosis of dogs with UC after medical treatment [32]. In our study, the major breeds were predominantly small breeds, particularly Dachshunds. This difference might be a result of the owners’ preferences in our country, where owners prefer small breeds, and the ownership rate of terriers is relatively low.

The limitations of this study include its retrospective nature and the fact that most patients did not receive histopathological diagnoses. The sensitivity of conventional PCR methods is lower than that of digital PCR. The detection of mutant genes is limited by the quantity of tumor genes in the liquid specimen. The mutated DNA may be diluted by inflammatory cells or normal epithelial cells, making it harder to detect and affecting the sensitivity for identifying the BRAF V595E mutation.

## 5. Conclusions

In conclusion, this study developed a conventional PCR method for detecting the BRAF V595E mutation in urine and prostatic wash fluid, facilitating the diagnosis of canine urothelial carcinoma (UC) and prostatic carcinoma (PC). The mutation detection rate in dogs with lower urinary tract or prostate masses was 54.1% in urine and 50% in prostatic wash fluid, with a 75% detection rate in confirmed UC or PC cases. This conventional PCR method offers a fast, reliable, and non-invasive screening test that aids in clinical decision making, especially when advanced equipment is unavailable.

## Figures and Tables

**Table 1:** Primers used to detect the canine BRAF gene (wild type and mutant) and the internal control.

PCR	Primer Pair	Sequences (5′–3′)	Size
Wild type (Wt)	cBRAF-F	GTAATGCTTGCTTTGCTAGGAA	138 bp
	cBRAF-Rw	CCCACTCCATCGAGATTTCA	
Mutant (Mu)	cBRAF-F	GTAATGCTTGCTTTGCTAGGAA	138 bp
	cBRAF-Rm	CCCACTCCATCGAGATTTCT	
GAPDH	GAPDH-F	ACCACAGTCCATGCCATCA	452 bp
	GAPDH-R	TCCACCACCCTGTTGCTGT	

**Table 2:** The detection rates of the BRAF V595E mutation in the tissues of canine urothelial carcinomas (UC) and prostate carcinoma (PC) samples, as well as in urine and prostatic wash specimens of dogs with urinary tract or prostate masses.

Specimens	Category	Number of Cases	Cases Passing the Internal Control	Cases with the BRAF Mutation	Mutation Rate
FFPE tissues		34	31	16	51.6% (16/31)
	UC	32	29	15	51.7% (15/29)
	PC	2	2	1	50% (1/2)
Liquid specimens of dogs with urinary tract or prostate masses		125	117	63	53.2% (63/117)
	Urine	116	109	59	54.1% (59/109)
	Prostatic wash	9	8	4	50% (4/8)

**Table 3:** The outcome of 41 dogs included in the follow-up study: 16 dogs were diagnosed with urothelial carcinoma (UC) or prostate carcinoma (PC), and 25 dogs did not undergo a histopathological examination after non-cancerous cytological results. The BRAF V595E mutation rate was 75% in these 16 dogs. The mutation-positive dogs (100%, 24/24) showed more tumor progression than did the mutation-negative dogs (35%, 6/17).

Follow-Up of 41 Dogs	One Year Follow-Up of the Mass or Lesion	*n*	BRAF V595E Mutation (n = 24)	No BRAF V595E Mutation (n = 17)
With pathological diagnosis		16		
	UC or PC; progression within 1 year	16	12	4
Without pathological diagnosis		25		
	Progression within 1 year	14	12	2
	Complete remission or stable within 1 year, without antineoplastic treatment	11	0	11

**Table 4:** The diagnostic method and presence of the BRAF V595E mutation in 16 dogs diagnosed with UC or PC after detection of the BRAF mutation in their liquid specimens.

Diagnostic Method	Diagnosis	*n*	BRAF V595E Mutation (n = 12)	No BRAF V595E Mutation (n = 4)
Cytology		16		
	Carcinoma in the urinary bladder	6	6	0
	Carcinoma in the prostatic gland	5	4	1
	Carcinoma in the urinary bladder and prostate	3	2	1
	No atypical epithelial cells *	2	0	2
Histopathology		4		
	Urothelial carcinoma	4	2	2

* These two dogs with non-cancerous urine cytological results underwent further histopathological examinations.

## Data Availability

The data presented in this study are available in this article.

## Supplementary Materials

The following supporting information can be downloaded at: https://www.mdpi.com/article/10.3390/ani14172535/s1, Figure S1: The original electrophoresis image of representative PCR results of the formalin-fixed paraffin-embedded tissues from canine urothelial carcinoma. PCRs using wild-type and mutant-type primer pairs were applied to detect the wild-type and V595E mutant-type of the BRAF gene in DNA extracted from six urothelial carcinomas (numbers 1 to 6). DNA extracted from a melanoma cell (M1) without the BRAF mutant gene and double-distilled sterile water were used as the wild-type BRAF gene and the negative control, respectively. DNA extracted from the urinary sediment of urothelial carcinoma with the BRAF mutation was used as a positive control.
