# Supplementary figures and images for: Diagnostic Value of Conventional Polymerase Chain Reaction for Detecting BRAF V595E Mutation in Liquid and Tissue Specimens of Canine Urothelial and Prostate Carcinomas
Source: Animals (Basel). 2024 Aug 31;14(17):2535. doi: 10.3390/ani14172535 (PMC11394148; doi:10.3390/ani14172535)

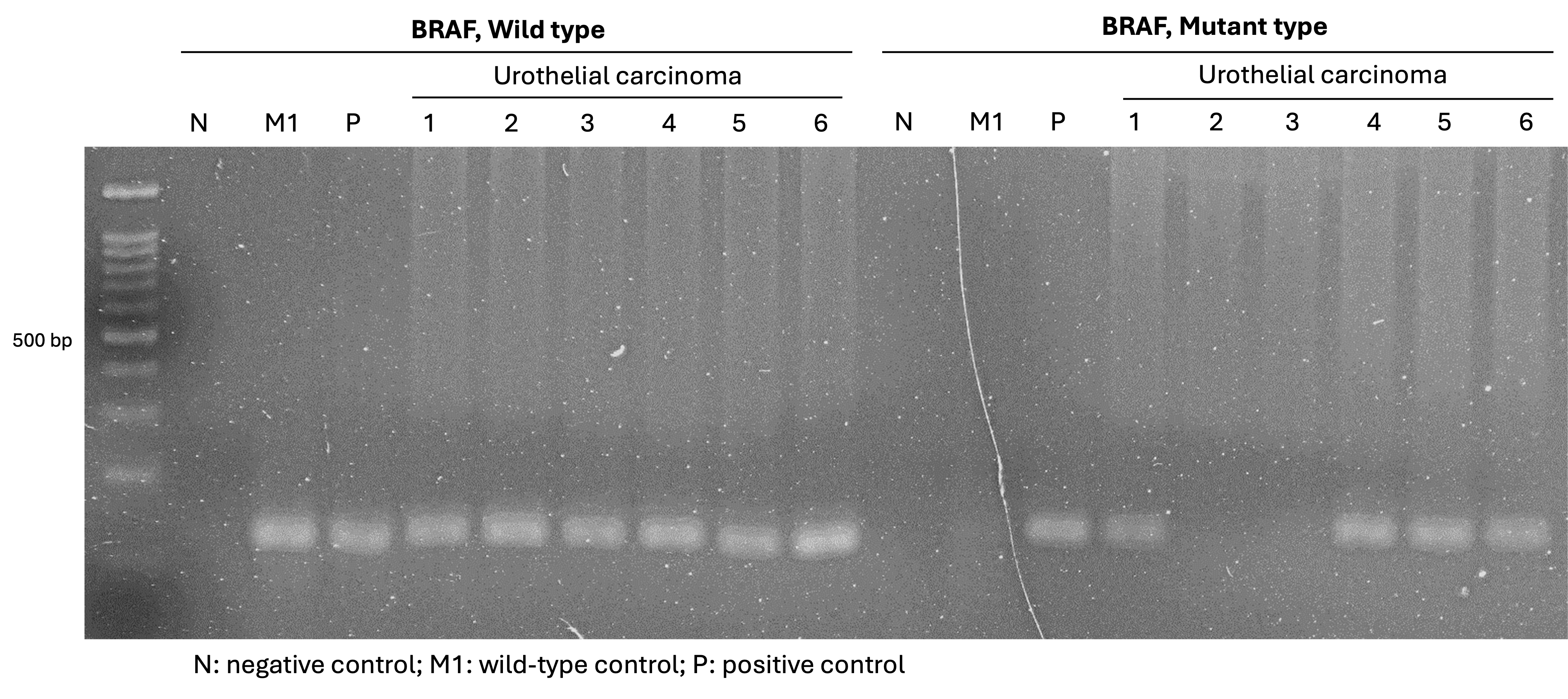

Supplement: Supplementary file 1 [file animals-14-02535-s001.zip › animals-3114038-supplementary.png]
